# Differentiation and Cell Survival of Myeloid Leukemia Cells

**DOI:** 10.1155/2012/370375

**Published:** 2012-07-31

**Authors:** George P. Studzinski, Geoffrey Brown, Michael Danilenko, Philip Hughes, Ewa Marcinkowska

**Affiliations:** ^1^Department of Pathology and Laboratory Medicine, UMDNJ-New Jersey Medical School, Newark, NJ 07103, USA; ^2^School of Immunity and Infection, College of Medical and Dental Sciences, University of Birmingham, Vincent Drive, Edgbaston, Birmingham B15 2TT, UK; ^3^Department of Clinical Biochemistry, Faculty of Health Sciences, Ben-Gurion University of the Negev, Beer Sheva 84105, Israel; ^4^Faculty of Biotechnology, University of Wroclaw, Tamka 2, Wroclaw, Poland

 The landscape of treatment for acute myeloid leukemia (AML) is currently a grim one. Apart from the AML subtype characterized by the 15:17 chromosome translocation known as acute promyelocytic leukemia (APL), which has shown lasting remissions when treated with all-trans retinoic acid (ATRA), especially when supplemented with the mildly toxic compound arsenic trioxide (ATO), mortality from the disease remains high. Thus, research into novel regimens of therapy is needed to supplement the current reliance on toxic compounds such as AraC and daunorubicin in the treatment of these diseases. Despite some spectacular clinical successes, ATRA-based differentiation therapy is not without its problems due to the induction of potentially life-threatening toxicities and the acquisition of therapeutic resistance in some patients. G. Brown and P. Hughes summarize the current state of knowledge in a comprehensive review entitled “*Retinoid differentiation therapy for common types of acute myeloid leukemia*” and suggest ways in which retinoid-based therapies can be improved by the inclusion of additional agents to increase the sensitivity of APL cells towards ATRA. This is followed in this issue by an example of cutting-edge research into the molecular basis for the efficacy of ATRA/ATO therapy for APL. B. Ozpolat et al. describe that at least part of the anti-APL effect of ATRA/ATO treatment can be explained by the inhibition of protein translation by these compounds. The report of these studies, entitled “*PKC*δ* regulates translation initiation through PKR and eIF2*α* in response to retinoic acid in acute myeloid leukemia cells*” also indicates that ATRA/ATO inhibit the PI3K/AKT/mTOR pathway, leading to an upregulation of the PKC delta/PKR axis. Both these articles add to our knowledge of the mechanism of ATRA in the treatment of myeloid leukemias and add to the debate on how this treatment may be improved. These new approaches may have importance outside the realms of leukemia treatments and may also lead to improved use of retinoids as therapies for other solid tumors.

Chronic myeloid leukemia (CML) has considerably better prognosis than AML, and as with APL, treatment can be targeted to a hybrid gene, here Bcr-Abl, which results from a reciprocal translocation between chromosomes 9 and 22. Specific kinase inhibitors, such as Imatinib, have been identified and offer front-line treatment for CML. Unfortunately, resistance to kinase inhibitors frequently develops, and G. N. de Moraes et al. in a review entitled “*The interface between BCR-ABL-dependent and -independent resistance signaling pathways in chronic myeloid leukemia*” analyze the known causes for this resistance and offer several feasible molecular targets, which may overcome the development of resistance to kinase inhibitors in CML.

The lessons from these two therapeutic successes are not easily transferred to other subtypes of myeloid leukemia, as the molecular lesions that can be attacked to eradicate the malignant cells appear to be “moving targets.” The mutations vary from AML case to case; secondary mutations appear and are often multiple, so current therapy largely depends on “brute-force” cell killing by administration of highly toxic agents, which have varying differential sensitivity for leukemic and normal cells. It seems, therefore, that alternate strategies are needed. The “by-pass the genetic lesion” suggested approach (G. P. Studzinski et al., PMID: 16046262) is based on the induction of cell differentiation by compounds such as vitamin D derivatives (VDDs). Here, instead of targeting a genetic lesion, the therapeutic agent induces the expression of transcription factors, which activate alternative but dormant routes to cell differentiation. Unfortunately, as pointed out by Harrison and J. A. Bershadskyi in a clinical perspective “*Clinical experience using vitamin D and analogs in the treatment of myelodysplasia and acute myeloid leukemia: A review of the literature*,” attempts to improve the therapy of AML with VDDs alone have not been successful so far. Further research on the mode of action of differentiation-inducing agents is, therefore, needed and the mechanisms involved should be explored in depth, to gain insights how to improve the differentiation regimens. One example of such exploration is provided in this issue by E. Gocek et al., who show multiple levels of regulation of the differentiation process in the report “*Regulation of leukemic cell differentiation through the vitamin D receptor at the levels of intracellular signal transduction, gene transcription, protein trafficking and stability*.” Another report on VDD action “*Cell-type-specific effects of silibinin on vitamin D-induced differentiation of acute myeloid leukemia cells are associated with differential modulation of RXR*α* levels*” by R. Wassermann et al. is more directly translational. This article addresses the potential pitfall of cell type specificity of biological responses. When attempts are made to enhance the effects of VDDs by combining them with an antioxidant silibinin, enhanced differentiation is seen in one AML subtype, but inhibition of differentiation is seen in another AML subtype. Advance knowledge of such antagonism is essential for future clinical trials.

An exciting recent development in our understanding of the molecular basis of myeloid disorders is the realization that small noncoding RNAs play a role in these diseases. Y. Yuan et al. summarize a variety of such recent data in an article “*MicroRNAs in acute myeloid leukemia and other blood disorders*.” It seems that the information these and other studies described in this issue will provide a beginning of a long road that will eventually lead to much improved therapy, targeted or not, for myeloid leukemia.

*George P. Studzinski* *George P. Studzinski* *Geoffrey Brown* *Geoffrey Brown* *Michael Danilenko* *Michael Danilenko* *Philip Hughes* *Philip Hughes* *Ewa Marcinkowska* *Ewa Marcinkowska*

